# Global and regional estimates of orphans attributed to maternal cancer mortality in 2020

**DOI:** 10.1038/s41591-022-02109-2

**Published:** 2022-11-20

**Authors:** Florence Guida, Rachel Kidman, Jacques Ferlay, Joachim Schüz, Isabelle Soerjomataram, Benda Kithaka, Ophira Ginsburg, Raymond B. Mailhot Vega, Moses Galukande, Groesbeck Parham, Salvatore Vaccarella, Karen Canfell, Andre M. Ilbawi, Benjamin O. Anderson, Freddie Bray, Isabel dos-Santos-Silva, Valerie McCormack

**Affiliations:** 1 International Agency for Research on Cancer, Lyon, France; 2 Program in Public Health and Department of Family, Population and Preventive Medicine, Stony Brook University (State University of New York), Stony Brook, NY USA; 3 Kilele Health Association, Nairobi, Kenya; 4 Centre for Global Health, US National Cancer Institute, Rockville, MD USA; 5 Department of Radiation Oncology, University of Florida, Gainesville, FL USA; 6 Makerere University, Kampala, Uganda; 7 Department of Obstetrics and Gynecology, School of Medicine, University of North Carolina, Chapel Hill, NC USA; 8 The Daffodil Centre, The University of Sydney, a joint venture with Cancer Council NSW, Sydney, New South Wales Australia; 9 Global Breast Cancer Initiative, Department of Non-communicable Diseases, World Health Organization (WHO), Geneva, Switzerland; 10 Department of Non-Communicable Diseases Epidemiology, London School of Hygiene and Tropical Medicine, London, UK

**Keywords:** Cancer, Social sciences

## Abstract

Despite women being disproportionally affected by cancer deaths at young ages, there are no global estimates of the resulting maternal orphans, who experience health and education disadvantages throughout their lives. We estimated the number of children who became maternal orphans in 2020 due to their mother dying from cancer in that year, for 185 countries worldwide and by cause of cancer-related death. Female cancer deaths—by country, cancer type and age (derived from GLOBOCAN estimates)—were multiplied by each woman’s estimated number of children under the age of 18 years at the time of her death (fertility data were derived from United Nations World Population Prospects for birth cohort), accounting for child mortality and parity-cancer risk associations. Globally, there were 1,047,000 such orphans. Over half of these were orphans due to maternal deaths from breast (258,000, 25%), cervix (210,000, 20%) and upper-gastrointestinal cancers (136,000, 13%), and most occurred in Asia (48%: India 15%, China 10%, rest of Asia 23%) and Africa (35%). Globally, there were 40 new maternal orphans due to cancer per 100,000 children, with a declining trend with a higher Human Development Index (range: 121 in Malawi to 15 in Malta). An estimated 7 million children were prevalent maternal orphans due to cancer in mid-2020. Accelerating the implementation of the World Health Organization’s cervical and breast cancer initiatives has the potential to avert not only millions of preventable female cancer deaths but also the associated, often-overlooked, intergenerational consequences of these deaths.

## Abstract (web summary)

According to 2020 estimates from 185 countries, Asia and Africa have the most maternal orphans due to cancer, with breast and cervical cancers responsible for almost half of maternal cancers.

## Main

Cancer is the second-leading cause of death worldwide, causing 5.5 million deaths in men and 4.4 million in women in 2020^1^. This mortality burden has an immense impact on patients, their families and health systems and societies, leading to over 170 million years of life lost and often catastrophic economic costs^2,3^. Less often considered is the extent and impact of parental deaths from cancer on their children. During the follow-up of a sub-Saharan African breast cancer cohort, we found that the number of children still under the age of 18 years at the time of their mothers’ death exceeded the number of breast cancer deaths, thus unveiling a common occurrence of orphans due to cancer in this setting^4^. As per the United Nations International Children’s Emergency Fund (UNICEF) definition, while these children are under the age of 18 years, they are maternal orphans, whereas children who have lost a father or both parents are referred to as paternal and double orphans respectively^5^.

The consequences of orphanhood from any cause of parental death can have a long-term impact on a child’s life in multiple domains, many of which are family, context and setting specific^6^. Maternal orphans have higher rates of mortality in childhood than their peers, both in low- and high-income settings^7,8^. As they grow up, orphaned children are at an increased risk of mental health disorders and suicide, as well as experiencing sexual violence^9–11^. Orphanhood is also associated with raised risks of teenage pregnancy, infectious diseases, including HIV/AIDS during adolescence and chronic diseases later in life^12^. In some settings, orphans are more likely to leave school prematurely and become entangled in a cycle of poverty^13^.

Overall, 71% of cancer deaths occur at the age of 60 years or older^14^, by which time most children of deceased adults are already aged 18 years or older; that is, at the time of death, most women last gave birth over 18 years ago. However, cancer deaths at younger ages can result in orphans. At these younger ages, both globally and in lower-income countries (LICs) in particular, women are disproportionately affected by cancer deaths compared to men^14^. This is primarily due to deaths from common cancers that predominantly or exclusively affect women, namely breast and cervix^15^. Additionally, cancer deaths in LICs are more likely to result in maternal orphans because LICs are characterized by younger populations and, hence, lower average age of cancer diagnosis^16^, as well as higher fertility and later maternal age at last birth^17^. Thus in LICs, if a women dies premanturely from cancer, her children are more likely to still be minors than in HICs.

In the present study, we aimed to fill the information gap on maternal orphans due to cancer. We focus on maternal orphans given the disproportionate burden of cancer deaths at young ages in women, the availability of high-quality fertility estimates and the central role of a mother in a child’s development, caregiving and education. Our aims were to provide country, regional and global estimates, for 2020, of the following: (i) the number of new maternal orphans due to cancer, for all cancers combined and by cancer site; (ii) risk of a child becoming a maternal orphan due to cancer; (iii) the number of new maternal orphans per 100 female cancer deaths; (iv) the number of prevalent (existing) maternal orphans due to cancer, by country; and (v) the age-distribution of new and prevalent maternal orphans due to cancer. We also examined variations of these estimates by a country’s place in the Human Development Index (HDI).

## Results

### Absolute burden of new maternal orphans due to cancer

**Fig. 1:**
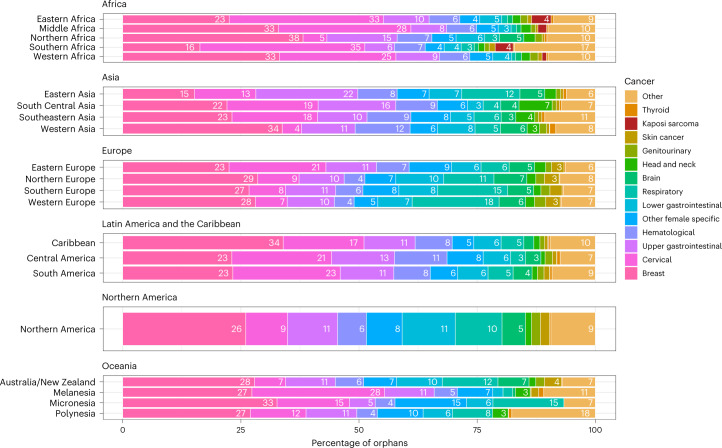
Percentage distribution of site-specific cancer deaths giving rise to new maternal orphans due to cancer in 2020, by region around the globe. Cancer codes (ICD-10) included in each of the 14 cancer groups are listed in Extended Data Table 3.

We estimated that the 4,404,000 cancer deaths in women in 2020 resulted in 1,047,000 new maternal orphans globally. Almost half (48%, 508,000) of these children were in Asia and over one-third in Africa (35%, 370,000), while Europe (60,000 orphans), Latin America and the Caribbean (76,000), Northern America (28,000) and Oceania (6,400) together comprised the remaining 16% (Table 1). The predominance of these orphans in Asia and Africa was driven by six countries, which comprised two-fifths of the worldwide total—India (157,000), China (107,000), Nigeria (53,000), Indonesia (42,000), Ethiopia (39,000) and Pakistan (38,000) (Supplementary Table 1). In terms of contributing cancer sites, deaths from breast cancer were the single largest cause of new maternal orphans globally (25%), followed by cervix (20%) and upper gastrointestinal (GI) cancers (13%, of which the majority were gastric or esophageal) (Table 2). The ranks of these top three cancers varied between regions and HDI categories, although breast cancer deaths always occupied first or second place throughout (Fig. 1 and Extended Data Table 1). In Eastern and Southern Africa, cervical cancer deaths led to more maternal orphans than breast cancer deaths. These two regions were also the only two where female deaths from Kaposi sarcoma contributed substantially (4% in each). In Eastern Asia, upper GI cancer deaths led to more maternal orphans than breast and cervical cancer deaths. In Europe, New Zealand and Australia, respiratory cancer deaths (majority lung) were the second-leading cause of maternal orphans (11–18%) after breast cancer, with the exception of Eastern Europe where cervical cancer deaths ranked second (21%). Lower GI deaths (dominated by colorectal cancer) contributed to 5% of maternal orphans globally but represented at least 10% in Australia, New Zealand, North America and Northern Europe.

**Table 1:** Global and regional distribution of new and prevalent maternal orphans due to cancer in 2020 and the ages of these orphans at the time of their mothers’ death

	Cancer deaths in women in 2020 (all ages)		New maternal orphans due to cancer in 2020							Prevalent maternal orphans due to cancer in 2020					
	No.	Average age at cancer death (years)^a^	No.	World’s total (%)	No. per 100 cancer deaths in women	No. per 100,000 children	Age distribution (years, row %)			No.	World’s total (%)	No. per 100,000 children	Age distribution (years, row %)		
							<5	5–9	10–17				<5	5–9	10–17
World	4,404,419	56	1,047,178	100	24	40	11	21	68	7,048,905	100	272	4	16	79
**By region**															
**Africa**	387,313		369,547	35	95	54				2,558,332	36	377			
Eastern Africa	134,037	51	154,332	15	115	66	13	24	63	1,082,846	15	460	5	18	77
Middle Africa	39,756	50	51,205	5	129	51	14	24	61	351,725	5	351	5	19	76
Northern Africa	87,046	54	39,688	4	46	39	13	24	63	264,460	4	262	5	19	76
Southern Africa	32,081	52	15,633	1	49	61	13	24	62	112,140	2	437	5	18	77
Western Africa	94,393	52	108,689	10	115	50	14	24	62	747,160	11	345	5	19	76
**Asia**	2,436,533		507,726	48	21	35				3,373,923	48	234			
Eastern Asia	1,419,391	58	119,893	11	8	32	9	18	73	731,981	10	194	4	15	81
South Central Asia	598,324	54	247,322	24	41	33	10	19	70	1,691,687	24	229	4	15	81
Southeastern Asia	315,298	55	99,548	10	32	45	10	20	70	671,395	10	301	4	15	81
Western Asia	103,520	55	40,962	4	40	40	11	22	67	278,859	4	276	4	17	79
**Europe**	870,658		59,801	6	7	38				370,856	5.3	235			
Eastern Europe	313,825	59	24,118	2	8	38	12	23	65	149,308	2.1	234	5	19	76
Northern Europe	128,775	60	8,065	1	6	33	9	20	71	50,823	0.7	209	4	16	80
Southern Europe	179,004	59	12,140	1	7	42	8	19	73	76,346	1.1	264	3	15	82
Western Europe	249,054	60	15,478	1	6	38	9	18	73	94,380	1.3	229	4	15	82
**Latin America and the Caribbean**	347,421		76,094	7	22	36				528,854	7.5	252			
Caribbean	28,806	56	5,416	1	19	40	10	20	70	37,256	0.5	274	4	15	81
Central America	64,527	54	19,710	2	31	31	11	20	69	138,247	2.0	214	4	16	80
South America	254,088	56	50,969	5	20	39	11	20	69	353,351	5.0	269	4	16	81
**Northern America**	331,325	59	27,553	3	8	31	9	18	73	173,072	2.5	192	4	14	82
**Oceania**	31,169		6,457	1	21	50				43,867	0.6	340			
Australia and New Zealand	25,998	59	2,394	0	9	31	10	20	70	14,977	0.2	195	4	16	80
Melanesia and Micronesia	4,867	54	3,950	0	81	78	13	23	64	28,157	0.4	559	5	17	78
Polynesia	304	55	113	0	37	64	8	18	74	733	0	414	3	14	83
**By HDI categories**^**b**^															
Very high HDI countries	1,549,055	59	129,026	12	8	35	10	20	70	822,198	12	226	4	16	80
High HDI countries	1,922,069	57	308,072	29	16	36	10	20	70	2,004,701	28	236	4	16	80
Medium HDI countries	666,966	54	319,241	30	48	37	11	20	69	2,193,349	31	257	4	15	81
Low HDI countries	238,244	51	280,310	27	118	55	14	24	62	1,952,392	28	384	5	19	76

^a^Among cancer deaths at ages 15–69 years. The children of women above these ages were adults in 2020. ^b^Ten of 185 countries/territories are not included as they do not have an HDI value. They represent 10,000 new maternal orphans due to cancer in 2020.

**Table 2:** New and prevalent maternal orphans in 2020 due to cancer deaths by 14 major cancers and the rest of the cancers (by total number, number per 100 cancer deaths in women, number per 100,000 children and the ages of orphans)

	Cancer deaths in women in 2020 (all ages)		New maternal orphans due to cancer in 2020							Prevalent maternal orphans due to cancer in 2020					
	No.	Average age at cancer death (years)^a^	No.	Percent of total	No. per 100 cancer deaths in women	No. per 100,000 children	Age distribution (years, row %)			No.	Percent of total	No. per 100,000 children	Age distribution (years, row %)		
							<5	5–9	10–17				<5	5–9	10–17
All cancer deaths	4,404,419	56	1,047,178	100	24	40	11	21	68	7,048,905	100	272	4	16	79
By cancer-related death															
Breast	682,288	55	257,561	24.6	38	9.9	11	22	67	1,724,459	24.5	66.6	4.2	16.4	79.4
Cervical	340,841	53	209,857	20.0	62	8.1	11	21	68	1,392,226	19.8	53.7	4.1	16.1	79.8
Upper gastrointestinal	955,243	58	135,962	13.0	14	5.2	9	19	72	841,825	11.9	32.5	3.7	14.8	81.5
Hematological	305,860	53	81,879	7.8	27	3.2	16	24	59	657,219	9.3	25.4	5.7	19.2	75.1
Other female-specific	328,930	57	63,054	6.0	19	2.4	9	19	72	395,339	5.6	15.3	3.8	15.1	81.1
Lower gastrointestinal	415,944	58	53,172	5.1	13	2.1	11	21	69	347,937	4.9	13.4	4.1	16	79.8
Respiratory	610,696	60	50,580	4.8	8	2.0	7	16	77	280,060	4.0	10.8	3.1	13.1	83.9
Brain and nervous system	112,711	53	34,704	3.3	31	1.3	15	23	62	265,564	3.8	10.3	5.3	18.3	76.4
Head and Neck	112,849	56	34,643	3.3	31	1.3	11	20	69	232,844	3.3	9.0	4.1	15.6	80.3
Genitourinary	116,709	59	12,750	1.2	11	0.5	11	20	69	82,075	1.2	3.2	4.2	16.1	79.7
Skin cancer	50,598	56	9,355	0.9	18	0.4	11	21	69	78,360	1.1	3.0	7.1	22.7	70.2
Kaposi Sarcoma	5,149	37	9,009	0.9	175	0.3	22	30	48	62,205	0.9	2.4	4.1	16	79.8
Thyroid	27,657	57	5,171	0.5	19	0.2	12	21	67	35,859	0.5	1.4	4.6	16.9	78.4
Other	338,944	54	89,482	8.5	26.4	3.5	14	23	63	652,934	9.3	25.2	5.2	18.1	76.7

^a^Among cancer deaths at ages 15–69 years. The children of women above these ages were adults in 2020.

**Fig. 2:**
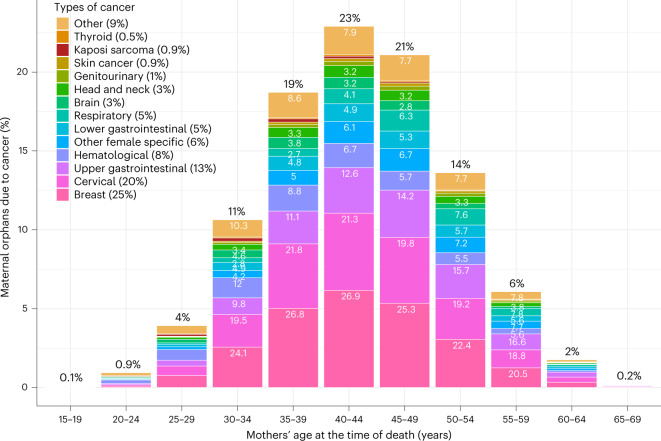
Percent distribution of maternal orphans due to cancer (worldwide) by mother’s age at the time of death and by type of cancer death within each category. Cancer codes (ICD-10) included in each of the 14 cancer groups are listed in Extended Data Table 3. Percentages on top of bars are % of maternal orphans due to cancer in each age group. Percentages within bars are % of maternal orphans arising from each cause of cancer-related death within each maternal age at death group.

**Extended Data Fig. 1:**
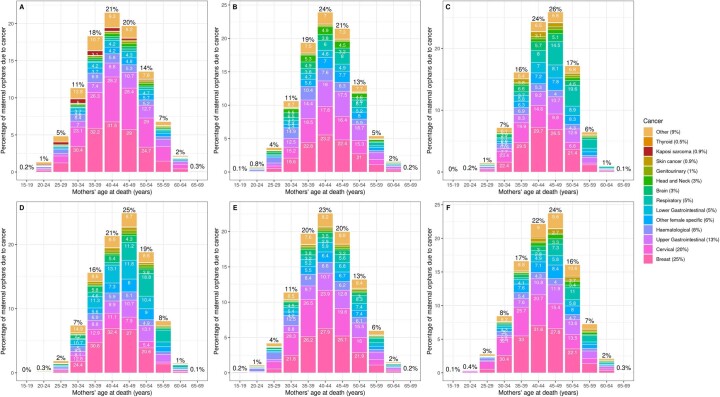
Distribution of maternal age at death when new maternal cancer orphans occurred, and of the type of cancer-related death within each age-at-death band. Data for six world regions: (**a**) Africa, (**b**) Asia, (**c**) Europe, (**d**) Northern America, (**e**) Latin American and the Caribbean and (**f**) Oceania.

In total, 63% of new maternal orphans arose from female cancer deaths at ages 35–49 years, with a mode at ages 40–44 years (Fig. 2), while 22% of orphaned children lost their mother when she was 50+ years and 16% under the age of 35 years. The contributing cancers to maternal orphans also shifted across these maternal age-at-death groups in each region, for example, with respiratory cancer deaths contributing to more maternal orphans for maternal deaths at older ages (Fig. 2 and Extended Data Fig. 1).

### Risks of maternal orphanhood

**Fig. 3:**
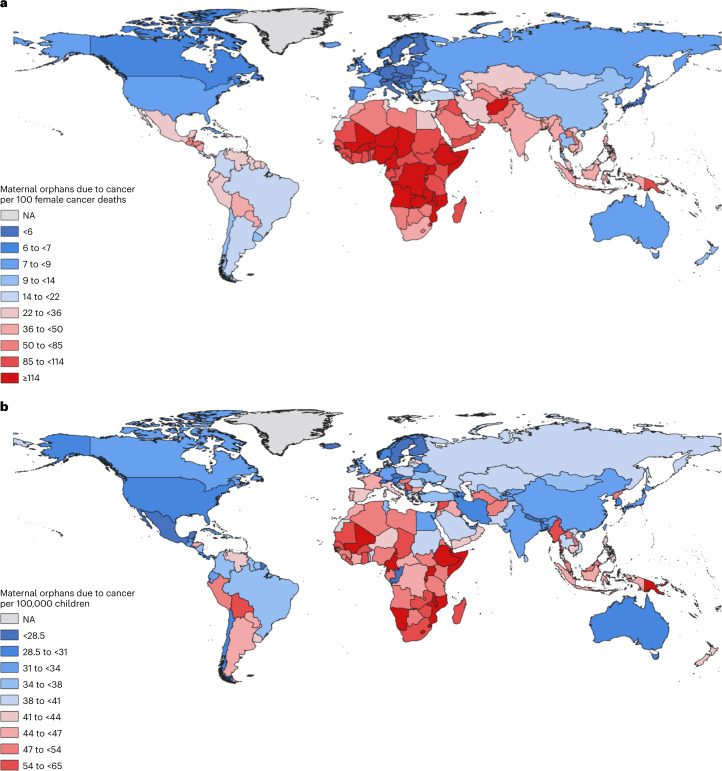
World map of new maternal orphans due to cancer in 2020. **a**, Number of new maternal orphans due to cancer per 100 cancer deaths in women in 2020. **b**, Number of new maternal orphans due to cancer per 100,000 children in 2020. NA: Not available.

**Extended Data Fig. 2:**
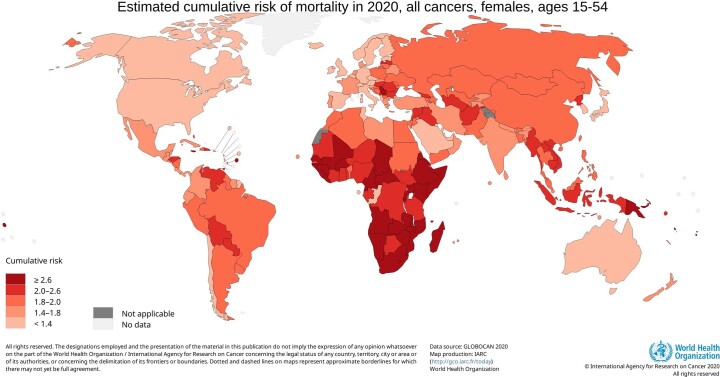
Cumulative risk (%) of death from cancer in women at ages 15–54 years (ages when 92% of maternal orphans due to cancer arise), 2020.

Apart from the total population size, the following two factors strongly influence a country’s total number of maternal orphans due to cancer. They are the average number of children under the age of 18 years per 100 female cancer deaths, largely reflecting past fertility (Fig. 3a), and the risk of cancer death among women at ages when most children remain under 18 years of age (i.e. deaths of women at ages 15–54 years which give rise to 92% of maternal orphans, Extended Data Fig. 2). The latter is a strong determinant of the number of maternal orphans due to cancer per 100,000 children, that is, a child’s risk of becoming a maternal cancer orphan independent of fertility rates (Fig. 3b).

**Extended Data Fig. 3:**
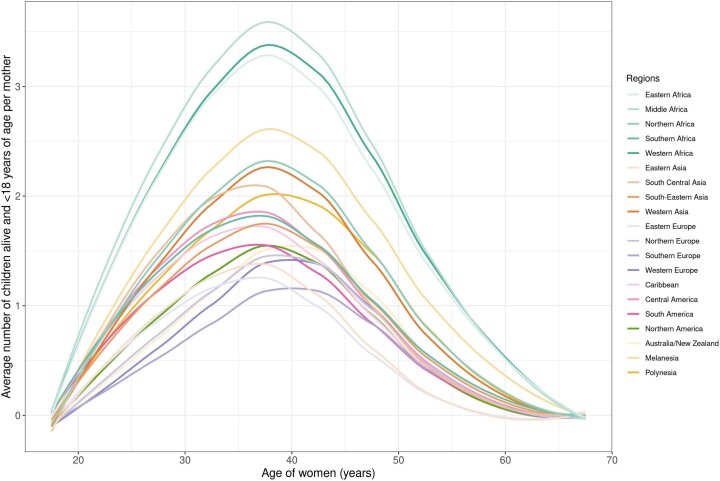
Mean number of living children < 18 years according to women’s age in 2020, by world region.

**Extended Data Fig. 4:**
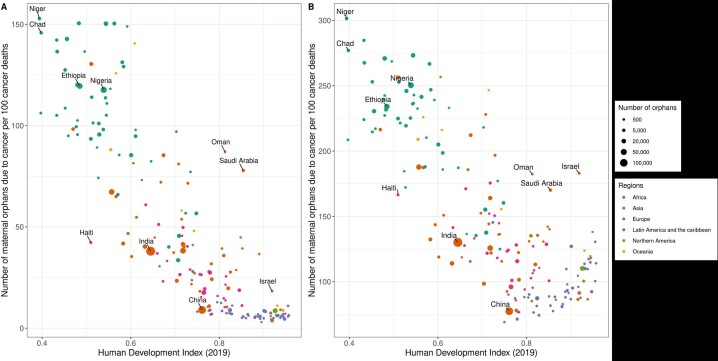
New maternal orphans due to cancer per 100 female cancer deaths by a country’s human development index for (A) cancer deaths at any age and (B) restricted to those occuring under age 50 years. 10 of 185 countries/territories are not included as they do not have a HDI value. They represent 10,000 new maternal orphans due to cancer in 2020.

The number of new maternal orphans per 100 female cancer deaths was the highest in Africa and other low-/middle-income countries (LMICs) (Fig. 3a) due to their high fertility rates (Extended Data Fig. 3). This was particularly the case in Middle, Western and Eastern Africa (average 3−3.3 children <18 years per woman aged 40–44 years), thus the ratio of new maternal orphans per 100 female cancer deaths was over 110 in these countries. Conversely, the ratio of new maternal orphans per 100 female cancer deaths was lower than ten in regions of low fertility - and often higher HDI - such as Eastern Asia (including Japan and China), Europe and Northern America (Fig. 3a). Although these differentials were partly influenced by the younger demographic of Africa and other LMICs, a strong inverse relationship between new maternal orphans per 100 female cancer deaths and a country’s HDI remained when restricted to deaths under age 50 (Extended Data Fig. 4).

**Fig. 4:**
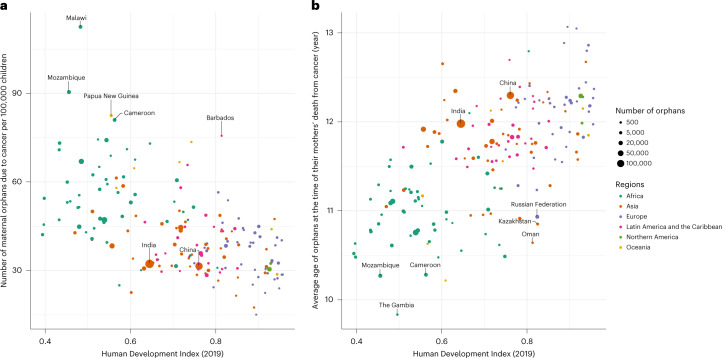
Number of new maternal orphans due to cancer per 100,000 children and the mean age at orphaning versus a country’s human development index (HDI). **a**, Number of new maternal orphans due to cancer per 100,000 children, in 2020, plotted against a country’s human development index. **b**, Average age at orphaning at their mother’s death, in 2020, plotted against a country’s HDI. Ten of 185 countries/territories are not included as they do not have an HDI value. They represent 10,000 new maternal orphans due to cancer in 2020.

**Extended Data Fig. 5:**
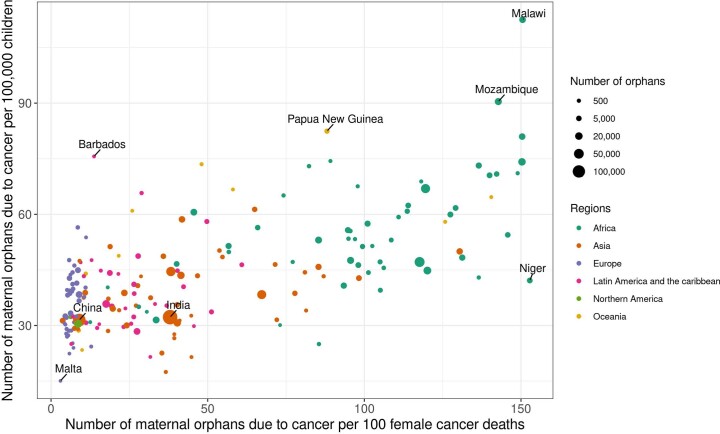
Number of new maternal orphans due to cancer per 100,000 children vs per 100 female cancer deaths for each country.

The 1,047,000 new maternal orphans globally translated to a global average of 40 orphans per 100,000 children. At a regional level, this number ranged from 15 in Malta to 113 in Malawi (Fig. 3b and Extended Data Fig. 5), but in 110 of 185 countries (59%) this number lay close to the global average, that is, between 30 and 50 orphans per 100,000 children (Extended Data Fig. 5). There was a general tendency for greater numbers of newly orphaned children per 100,000 children in countries with lower HDI (Fig. 4a), driven, for the most part, by the risk of cancer death in women at ages 15–54 years, which was highest in parts of Africa, followed by some Asian and South American countries (Extended Data Fig. 2). Indeed, of the 10% of countries with a ratio over 65 new maternal orphans per 100,000 children, 14 were in Africa (in descending order: Malawi, Mozambique, Cameroon, Djibouti, Uganda, Mali, Namibia, Equatorial Guinea, Burundi, Somalia, Comoros, Eswatini, Ethiopia and Lesotho), two in the Caribbean (Barbados and Jamaica) and three in Oceania (Papua New Guinea, Fiji and Samoa; Supplementary Table 1). In most of these listed African countries, as well as in Papua New Guinea and Fiji, cervical cancer mortality rates at ages 15–54 years were between two and eight times higher than the global average. Each of these countries also had high fertility rates. In contrast, in Jamaica (612 new maternal orphans in 2020) and Barbados (51 new maternal orphans), fertility rates and thus maternal orphans per 100 cancer deaths were not particularly high, but breast cancer mortality rates at ages 15–54 years were three to four times the global average. Outside of this top decile, other countries that had a high number of orphans per 100,000 children relative to other countries in their region were Serbia (56 new maternal orphans per 100,000 children) and Montenegro (54) in Europe—where female cancer deaths at ages 15–54 were dominated by breast, cervix and lung—Syria (61) and Myanmar (59) in Asia and Bolivia (58) and Peru (49) in South America. Conversely, there were several countries, such as Nepal, Niger and Bangladesh, where the number of new maternal orphans per 100 female cancer deaths was high, but the absolute risk of becoming a maternal orphan per 100,000 children was relatively low or moderate, due to the low/moderate cancer mortality rates in women. Finally, India and China make interesting contrasts. Both had similar numbers of new maternal orphans per 100,000 children (32), resulting from similar rates of female cancer mortality at ages 15–54 years, but India had 50% more orphans (157,0000) than China (107,000) due to its higher fertility rate and much younger population structure.

### Age of children at orphaning

Most newly orphaned children were aged 10 years or over at the time of cancer bereavement (69%), 21% were aged 5–9 years and 11% were aged under 5 years (Table 1). The mean age of new maternal orphans for most countries (166 of 185 countries, 90%) varied between 10.5 and 12.5 years, with a clear positive relationship of older age at orphaning in countries with higher HDI (Fig. 4b and Table 1). Note that these differences in age at orphaning by HDI were relatively small compared to international differences in age at first/last birth because many of the children born to younger mothers will be adults before a potential maternal cancer death (for example, a child born to a 20-year-old mother is an adult when the mother is 38 years and age of cancer diagnosis and thus potential cancer death is most often over age 38 years). These differences imply that, although 35% of all maternal cancer orphans occurred in Africa, this percentage was higher (43%) among the subset of these children who were under the age of 5 years at orphaning.

### Prevalent maternal orphans due to cancer

The number of prevalent maternal orphans due to cancer in mid-2020 was estimated to be 7,048,000. The geographic distribution (Table 1) was similar to that of new maternal orphans but prevalent orphans were older—approximately 4% were aged under 5 years, 16% were aged 5–9 years, and 79% were aged over 10 years (Table 1). Sensitivity analyses revealed that this estimate of 7,048,000 varied between 6,615,000 and 7,423,000 (Extended Data Table 2). The scenarios considered for the impact of maternal death on subsequent child survival only marginally altered the prevalence estimate (reducing the global prevalence by up to 90,000 (<1.5%)), whereas models with varying historic cancer mortality rates altered the estimates by up to ±375,000 (5.3%).

## Discussion

The global cancer burden can be evaluated from many different perspectives, which together help to inform cancer policy and resource allocation along the continuum of cancer prevention and care. To date, the intergenerational consequences of the cancer burden have been afforded scarce attention. We therefore provide global, regional and national estimates of maternal orphans due to cancer in this study. Our estimate of approximately 1 million new maternal orphans due to cancer in 2020 and a point prevalence of 7 million maternal orphans due to cancer implies that cancer deaths were responsible for approximately 15% of prevalent maternal orphans in 2020^18^. The disaggregation of maternal orphans by cause of cancer-related death, country and HDI highlights further dimensions of global health inequities, as well as the preventability of many of the cancer deaths contributing to the vast number of maternal orphans due to cancer worldwide.

The two cancer-related deaths causing almost half of maternal cancer orphans are the female-specific cancers of breast and cervix. By no coincidence, these are the two cancers for which the World Health Organization (WHO) has launched targeted initiatives in the form of the Global Breast Cancer Initiative^19^ (in 2021) and the Cervical Cancer Elimination Initiative^20^ (in 2020). The scale of the maternal orphans resulting from these two cancers provides yet another incentive for investment into and accelerating scale-up of these programs. Action is especially needed in Asian, African and other countries where orphaning at young ages is more common. For cervical cancer, screening and treatment of cervical intra-epithelial neoplasia in adult women will greatly reduce cervical cancer mortality rates, whereas human papillomavirus (HPV) vaccination of young girls before the onset of sexual activity will protect the future generations of women from cancer and thus their children from orphanhood^21^. While this outlook is promising, the situation of averting breast cancer deaths is more complex. Incidence rates of breast cancer at postmenopausal ages are rising as fertility and lifestyle transitions continue^22^, and any subsequent deaths would lead to older maternal orphans. Reducing breast cancer-associated maternal orphans will require improvements in breast cancer survival in LMICs toward the high levels that have been achieved in high-income countries (HICs)^23^. The diagnosis of breast cancer at early stages coupled with timely quality treatment will be essential components herein, for which an immense investment into cancer diagnostics, pathology, surgery, chemotherapy and radiotherapy facilities, and professional training is needed. The third contributor to maternal cancer orphans globally is upper-GI cancers, mostly due to deaths in Asia and Africa. These include cancers such as those of esophagus, stomach and pancreas, for which survival has remained poor everywhere, even in HICs, and which have been granted little research funding or attention relative to their global mortality burden^24,25^.

From a social inequalities perspective, cancer often displays complex divergent patterns by cancer site and between incidence and mortality rates^26^, which are revealed once again in the multiple influences on maternal orphans. At the relevant ages (15–54 years), women living in countries with higher HDI have three to four times higher risks of cancer (all sites) than their counterparts in countries with lower HDI. Yet this relationship reverses when the risk of cancer death at the population-level is considered. Indeed, women in countries with lower HDI have a high risk of cancer deaths owing to lower patient survival rates. Thus, maternal orphans due to cancer were highest in low HDI countries, both in terms of orphans per 100,000 children and per 100 female cancer deaths. Our previous work in the African ABC-DO breast cancer cohort illustrated further within-country inequities in the risk of maternal orphans based on the real-life experience of over 2,200 women. Those with lower educational levels were diagnosed with breast cancer at later stages, were less likely to receive treatment and thus had lower survival rates than women with higher educational levels^27–29^. They also had more children, who thus became maternal orphans^4^. In that study, similar to the global estimates presented here, 17% of the maternal orphans were under age 5 at the time of maternal death. An intergenerational cycle of poverty and disadvantage is thus perpetuated as families are often left impoverished through catastrophic financial expenditure during cancer care^4^. Orphans are less likely to complete school, more likely to experience poverty, and more likely to have substantial health challenges across their life course than non-orphaned children^7,8,11,13,30^. Maternal orphans due to cancer thus need to be tackled within the realm of SDG10 to ‘reduce inequality within and among countries’.

In 2020, there were approximately 147 million prevalent orphans from any cause^18^ and approximately 16 million children were newly orphaned. Far more orphans have lost a father (58%) than a mother (31%) and 11% are double orphans. The major causes of orphaning include HIV, natural disasters, wars, chronic poverty, chronic diseases or disease outbreaks such as coronavirus disease 2019 (COVID-19)^12,18^. Cancer as a cause of orphaning has not been investigated in great detail in terms of its impact on children, but it might confer unique risks. For example, unlike sudden deaths, the catastrophic financial toxicity of a failed cancer treatment can leave families struggling to provide for the needs of children^4^. A parental cancer death may also be associated with a real risk in the future, or stigma-related perceived risk, of cancer in the orphan. The HIV field has long estimated orphanhood due to HIV/AIDS as a key measure of the social impact of the epidemic. In that context, the staggering figures have been a catalyst for integrating child-centered responses into the HIV response. For example, the President’s Emergency Plan for AIDS Relief (PEPFAR) allocates 10% of its budget toward programming for orphans and vulnerable children^31^. Although maternal orphans due to cancer and HIV have many differences—orphans due to HIV are much younger, are concentrated in sub-Saharan Africa, many were HIV-positive themselves in the early stages of the HIV epidemic and are often double orphans, which would be rare for orphans of cancer^5^—a lot that has been learned from the support needed for orphans due to HIV is relevant to all orphans. To avoid stigmatizing children, most programs serve those who have been orphaned by any cause, which would include those orphaned due to cancer. The current estimates indicate that many HIV-endemic countries are indeed grappling with large numbers of maternal orphans due to cancer^32^. These observations agree with our estimates, as the highest corresponding risks were in East and Southern Africa. Indeed, some orphans of HIV are also orphans of cancer due to the many HIV-defining malignancies^33^. Most orphans will and should be taken in by surviving parents or extended family within the community, as in the context of HIV^34^. Strengthening the family’s capacity for caregiving through cash transfers, parenting interventions and psychosocial support are promising support strategies^30,35^. For cancer specifically, some cancer foundations such as the Lalla Salma foundation in Morocco also support the education costs of orphans of cancer^36^. The age distributions of new and prevalent orphans at the time of their mothers’ death, which were remarkably similar across the different world regions, highlight the need to ensure that such strategies will cater to the varying age-related needs of the orphans, stretching from very early childhood to late adolescence.

This study has some limitations. Our estimates required several explicit assumptions and reliance on global databases. GLOBOCAN includes worldwide estimates but in some countries, and particularly in LMICs, complete coverage of cancer deaths was not available or was of limited quality, thus interpretations should be made with caution^37^. Analyses at a country level were aggregated to provide the totals worldwide, which meant 99.4% of global cancer deaths in women were included (as some small territories included in global totals but do not appear as individual countries). Thus uncertainty intervals around maternal cancer orphan estimates would be driven largely by uncertainties in the GLOBOCAN estimates, which are provided on the Global Cancer Observatory, ‘Cancer Today’ website (https://gco.iarc.fr/). At the 95% level, they extend ±3.6% around the best estimates for global female cancer deaths (all ages), but with regional variations in ascending order as follows: ±0.5% for Northern America, ±2.8% for Asia, ±4.0% for Europe, ±4.6% for Latin America and the Caribbean, ±10% for Oceania and ±22.6% for Africa. We also focussed the detailed results on new rather than prevalent maternal cancer orphans because the latter requires additional assumptions. Indeed, GLOBOCAN estimates are for a single year (2020), whereas historic data are only available for a small subset of countries. We thus had to make assumptions about the number of cancer deaths from 2003 to 2019. Nevertheless, we provided sensitivity analyses of prevalence estimates based on different scenarios of all-cancer mortality trends and showed that the prevalence estimate of 7 million is robust to the order of ±0.4 million. Assuming that past trends were stable may underestimate prevalent orphans because, as can be seen on IARC’s Global Cancer Observatory’s *Cancer Over Time* data, mortality rates have been declining in the past 15 years for the countries (mostly high-income) where data are available^38^. Further, the cancer-site-specific breakdown of prevalent maternal cancer orphans is likely to be less reliable than for new orphans. An additional consideration for the 2020 prevalent orphan estimates was the subtraction of children who died as a result of their mothers’ death, i.e. between their mother’s death and mid-2020^8,39^. We illustrate that the estimates were robust to a range of relevant mortality rate ratios applied, from 1.25 to 2.6. These ratios were selected to match the ages of maternal orphans due to cancer, which are typically much older than orphans from other causes (89% of maternal orphans of cancer were orphaned aged 5 years or older). In addition, estimates of the past fertility of women incorporated the associations between reproduction and cancer risks for three cancers, but beyond this adjustment, we assumed that the fertility history of women who died from cancer (at ages when orphans are possible) did not differ from that of their birth cohort. This assumption may lead to a slight over-estimation of orphans because cancer treatment affects subsequent fertility. This effect is not likely to be as large as cancer deaths at younger ages are concentrated in LMIC settings where median survival times are short (approximately 3 years)^28,40^, that is too short a period to alter a woman’s average fertility. However, future estimates would benefit from estimating the time-since-diagnosis among a cross-section of women who die from cancer and incorporating a reduced or zero fertility in the postdiagnosis period, especially for cancers such as ovarian and endometrial where treatment may involve removal of the uterus and ovaries. Such modifications may affect estimates of maternal orphans in settings where survival rates are high and thus deaths may be many years after the original cancer diagnosis. Considerations of the availability of fertility-preservation treatments in some settings will also be pertinent. Overall, we note that—with some assurance—our estimates for Africa were very similar to those observed in the ABC-DO cohort study^4^.

This study provides partial insight into the orphans resulting from cancer deaths as we have only estimated the orphans due to cancer deaths in women and not the corresponding orphans due to cancer deaths in men. The latter estimates are more complex to make because of the need to first estimate male fertility rates, for which several methods are proposed^41^. The cancer deaths that give rise to paternal orphans are expected to differ greatly from those for maternal orphans, not only in terms of contributing cancers but also because of the longer time span when men can reproduce. While the female reproductive life is assumed (in national fertility data) to span from 15 to 49 years, it can extend up to 70 years of age for men, that is ages when cancer incidence and mortality rates are substantially higher. Thus, for a comprehensive assessment, it will be imperative to extend to estimates of the number of paternal orphans due to cancer and to examine the impact on children’s lives after the loss of either parent due to cancer.

In conclusion, we have revealed that children are affected on large scale by the loss of their mothers due to cancer globally. This impact reveals yet another reason for the urgency of cancer control plans, propelling programs to prevent cancer and cancer deaths in the first place. Alongside these efforts, support to families and communities caring for orphaned children is needed to ensure that these children receive the same opportunities, education and good health as other children worldwide.

## Methods

Our working definition of a maternal orphan due to cancer is a child under age 18 whose mother has died from cancer. Cancer deaths were considered to be deaths from all malignancies (International Classification of Disease Tenth revision (ICD-10) C00-97). For 2020 estimates, new maternal orphans were orphaned in 2020, while prevalent maternal orphans were children under age 18 in mid-2020 who became maternal orphans at any time in the past. Central to all estimates of these orphans are the estimates of new orphans in a particular year. For a brief overview of the methodology, to estimate the absolute number of these orphans, we adopted methods similar to that for COVID-19-orphans^12^, that is using the number of female cancer deaths, by cancer site, at a given age at death and in a given country as the starting point. We multiplied these deaths by the estimated average number of children expected to be alive in 2020, per woman, at the age the woman died. The number of children was assumed to be a function of the age-specific fertility rates of that woman’s birth cohort in the prior 18 years, adjusted to the specific cancer death in question (details below) and additionally accounting for the probability that the child survived in 2020 (using the country-age-specific child mortality rates as the child advances from birth to 2020).

### Data sources

We needed country-level data on three items: (a) the absolute number of female cancer deaths in 2020, (b) fertility rates in women during 2002–2019 and (c) mortality rates of children during 2003–2020.

- Estimates of the number of female cancer deaths in 2020 were sourced from GLOBOCAN^14,42^. These data were obtained in 5-year age groups (0–4, 5–9,…, 85+) for 185 countries/territories (simply called ‘countries’ in the rest of the manuscript) and 35 exhaustive and mutually exclusive cancer sites (listed in Supplementary Table 1). In brief, the GLOBOCAN estimates were assembled at the national level using the best available sources of cancer incidence and mortality data for a given country, with priority given to short-term projections of incidence and mortality rates^42^. In instances where country data were not available, GLOBOCAN extrapolates from neighboring countries’ data. Of the 185 countries, 2020 mortality data are most accurate for 80 countries, being based on observed national mortality rates projected for 2020. For 21 countries, recent national or regional mortality rates were applied to the 2020 population, whereas for 81 countries, mortality rates were modeled based on incidence-to-mortality ratios derived from registries in neighboring countries (models are specific to regions: Western Asia, sub-Saharan Africa, Northern Africa, Oceania, South Central Asia and Southeastern Asia) and finally in three countries rates were estimated as an average of those from selected neighboring countries.
  Mortality data were obtained for 35 topographic cancer sites, which were then categorized into 13 groups for reporting purposes. Their codes in the ICD-10 and grouping are provided in Extended Data Table 3. This grouping was exhaustive and mutually exclusive.
- Fertility rates in women, that is the average number of births per 1,000 women, were extracted from the 2019 revision of the United Nations (UN) World Population Prospects, which are available for 201 countries/territories with at least 90,000 inhabitants^43^. The UN countries and the composition of geographical areas follow those in ‘standard country or area codes for statistical use’ WPP (ST/ESA/STAT/SER.M/49/Rev.3). Fertility rates for each country are based on a combination of birth histories from Demographic and Health Surveys, census data, reproductive and health surveys, multiple indicator cluster surveys and intercensal demographic surveys. The published fertility rates correspond to 5-year age groups from ages 15−49 years (outside of which fertility is zero) and 5-year calendar periods (2000–2019), running from 1 July to 30 June of the initial and final years. Note that because UNWPP fertility rates are zero at age 50 years and over, for all women who die at age 68 or over, these deaths cannot give rise to maternal orphans.
- Mortality rates from ages 0−17 years during each of the years from 2003 to 2020 were also extracted from UNWPP for the same 201 territories^43^.

In total, 185 countries, for which cancer mortality estimates are available in GLOBOCAN, were included in the present estimates of maternal cancer orphans. This differs from the 201 territories/countries in the UN data, due to two UN listings (Hong Kong and Macau) being included within China in GLOBOCAN and 14 UN listings for which GLOBOCAN estimates are not available (Antigua and Barbuda; Aruba; Channel Islands; China, Taiwan Province of China; Curacao; Grenada; Kiribati; Mayotte; Micronesia (Fed. States of); Saint Vincent and the Grenadine; Seychelles; Tonga; United States Virgin Islands; and Western Sahara). For all regional distributions presented, Micronesia (13 new maternal orphans due to cancer) and Polynesia (114 new maternal orphans due to cancer) in Oceania were combined due to the small number of maternal orphans due to cancer. We also examined variations in the number of new maternal orphans (per 100 female cancer deaths and per 100,000 children) by a country’s 2020 HDI. HDI for a country reflects a composite measure of a nation’s longevity (life expectancy at birth), education (mean years of education of children and of adults) and income (gross national income per capita). Of the 185 countries/territories included in this analysis, 175 had an HDI value in 2020 (all except French Guyana, French Polynesia, Guadeloupe, Guam, Democratic People Republic of Korea, Martinique, New Caledonia, Puerto Rico, La Reunion and Somalia). In 2020, HDI values ranged from 0.39 in South Sudan to 0.96 in Norway. HDI categories are low (<0.55), medium (0.55–0.69), high (0.70–0.79) and very high (0.8–1.0).

### Statistical analysis

#### New maternal orphans due to cancer in 2020

Calculations of new maternal orphans due to cancer in 2020 were made at the level of country, age at death and cause of cancer death. We denote NMC_2020,*c*,*t*,*md*_ as the number of new maternal orphans due to cancer (NMC) occurring in 2020 in country *c* due to cancer deaths from cancer-related death type *t* in women who died at age *md*. The total number of new maternal orphans due to cancer in a country is the sum of NMC_2020,*c*,*t*,*md*_ across all the ages at cancer death which can give rise to NMCs and across the 35 topographical cancer types: $\mathrm{NMC}_{2020,c} = \mathop {\sum }\nolimits_{t = 1}^{t = 35} \mathop {\sum }\nolimits_{md = 15}^{md = 67} \mathrm{NMC}_{2020,c,t,md}$. NMC_2020,*c*,*t*,*md*_ was estimated by multiplying the number of deaths from cancer type *t* in women who died at age md in country *c* in 2020 (D_2020,*c*,*t*,*md*_) by the average number of living children (per woman, A_2020,*c*,*md*_) who were aged under 18 for the corresponding cohort of women who died from cancer type *t* at age *md* in country *c* in 2020: NMC_2020,*c*,*t*,*md*_ *= D*_2020,*c*,*t*,*md*_ x *A*_2020,*c*,*md*,*t*_*. D*_2020,*c*,*t*,*md*_ (cancer deaths for a single year of age at death) were calculated from the number of deaths in the corresponding 5-year age-at-death category, divided by five.

The average (per woman) number of children under 18 years in 2020, *A*_2020,*c*,*md*,*t*_ is a function of the annual fertility rates between mid-2002 and mid-2019 of the woman’s birth cohort (that is with year of birth (2020-*md*)), multiplied by the probability that the child survived until their mothers’ death in 2020.

$$A_{c,md,t} = \mathop {\sum }\limits_{y = 2002}^{y = 2019} \mathrm{RR}_t\,x\,\mathrm{FR}_{c,y,ma}\,x\,P_c(\mathrm{alive}_{2020}|y)$$

Here

(i) FR_*c*__,*y*,*ma*_ is the fertility rate (per woman) in country *c* in calendar year *y* for women at age *ma*, where *ma* = *md* − (2020 − *y*) and FR_*c*,*y*,*ma*_ = 0 if *ma* > 50;
(ii) *P*_*c*_(alive_2020_*|y*) is the probability that a child born in year *y* in country *c* is alive in 2020. This probability was calculated using each country’s lifetables as follows:
  $$P_c\left( {\mathrm{alive}_{2020}{{{\mathrm{|}}}}y} \right) = {{{\mathrm{exp}}}}\left\{ { - \mathop {\sum }\limits_{x = 0}^{x = 2019 - y} \mathrm{Mx}_{c,\,y + x}} \right\}$$
  where *Mx*_*c,y+x*_ is the mortality rate at age *x* in country *c* in year *y*+*x*, that is year of birth + *x* years;
(iii) RR_*t*_ incorporates an adjustment to account for the fact that, for women who died of cancers where parity is a known risk factor, fertility would differ from the average fertility of her birth cohort and therefore, the average fertility rates needed correcting. This adjustment was to multiply the average number of children in a woman’s birth cohort by RR_*t*_, where RR is the relative risk of the association of parity with the risk of the specific cancer *t* (per unit increase in parity). RR_*t*_ was assumed to be 1 for all cancer types, except cervix (RR=1.1), ovary (RR=0.80) and breast cancer deaths over the age of 50 years (RR=0.93)^44^. These RRs were obtained from high-quality meta-analyses or large studies, which assessed the associations of parity with risk of the specific cancer type. High parity increases and decreases the risk of cervical and ovarian cancer, respectively, thus for these cancer deaths, in accordance with their parity-risk associations, the adjustments imply that the average number of children was increased by 10% for women who died from cervical cancer^45^ and reduced by 20% for ovarian cancer deaths^46^. For breast cancer risk, high parity is inversely associated with breast cancer risk at postmenopausal ages only (RR 0.93); however, at premenopausal ages, when most maternal orphans arise, it is at most weakly associated, thus we only made an adjustment for breast cancer deaths over the age of 50 years^47^. For other cancer sites, RRs were 1, because parity is not an established risk factor for upper GI cancers (gastric, esophageal), or lower GI cancers (colorectal), thus no adjustment of national fertility rates was needed. No adjustments were made for lung cancer as meta-analyses did not establish an association but rather observed large between-study heterogeneity^48,49^.

#### Prevalent maternal orphans due to cancer in 2020

As an overview, the point prevalence of maternal orphans (PMC_*c,t*_) in country *c* due to cancer death *t* was made for mid-year 2020, that is the number of children under the age of 18 years on 1 July 2020, who had lost their mother due to cancer at any time in the past. This prevalence was estimated as the sum of new maternal orphans due to cancer for female cancer deaths across the year of death (*yd*) for each year from mid-2003 to mid-2020, as described above, minus any children who were estimated to have died before mid-2020. Prior deaths were estimated using the country’s age-specific mortality rates *Mx* up to the maternal death, and thereafter by applying a higher mortality rate than that in national lifetables (MRR_MC_ *x* Mx) because studies across diverse settings have observed increased mortality of children who had lost a parent to any cause, including to cancer. The magnitude of this effect may vary between settings. In Nordic countries, children of parents who died from cancer have 1.25 times higher mortality than their peers, while in Bangladesh, mortality rates were 2.6 times higher at ages 2−10 years^7,8^, that is at the ages when 95% of new maternal orphans due to cancer occur. Thus, we applied the following range of mortality rate ratios associated with being a maternal orphan due to cancer (MRR_MC_): (a) 1.25 (considered a minimum), (b) 1.5, (c) 2.0, (d) 2.60 (maximum) and (e) as a comparator to gauge the total effect, an MRR_MC_ of 1.0 (that is, motherless children have the same mortality rates as their peers).

$$\mathrm{PMC}_{c,t} = \mathop {\sum }\limits_{yd = 2003}^{yd = 2019} \mathop {\sum }\limits_{md = 15}^{md = 67} D_{yd,c,t, {md}}\left( {\mathop {\sum }\limits_{y = 2002}^{y = \mathrm{yd}} \mathrm{RR}_t\,x\,\mathrm{FR}_{c,y,ma}\,x\,P_c( \mathrm{U}18_{2020}|y,yd)} \right)$$

Here

(i) *c* = country, *md* = woman’s age at death, *yd* = woman’s year of death, *y* = year of birth of child;
(ii) (*D*_*yd*,*c,t*,*md*_) is the number of deaths in year *yd*, in country *c*, from cancer type *t*, in women who died at age *md*;
(iii) RR_*t*_ is an adjustment factor for the association between parity and risk of cancer, for cancer types where parity is a known risk factor (see above);
(iv) FR_*c*,*y*,ma_ is the fertility rate (per woman) in country *c* in calendar year *y* for women at age *ma*, where *ma* *=* *yd* − (2020 − *y*);
(v) *P*_*c*_(U18_2020_*|y,yd*) is the probability that a child born in year *y* whose mother died in year *yd* in country *c* is alive and under 18 years of age in 2020:

$$P_c\left( {\mathrm{U1}8_{{\it{\mathrm{2020}}}}|y, yd} \right) = 0\,\mathrm {if}\,2020 - y \ge 18;$$

otherwise, the probability was calculated with different mortality rates before and after maternal death in year yd as follows:

$$P_c(\mathrm{U}18_{2020}|y,yd) = {{{\mathrm{exp}}}}\left\{ { - \mathop {\sum }\limits_{x = 0}^{x = yd - y} Mx_{c,yd} - \mathrm{MRR}_{\mathrm{MC}}\mathop {\sum }\limits_{x =yd- y + 1}^{x = 2020} Mx_{c,\,y + x}} \right\}$$

For the prevalent estimates of maternal orphans due to cancer, we needed the number of cancer deaths in women during 2003–2019, which could not be taken from previous GLOBOCAN releases because the estimation methodology changes over time. Thus for compatibility with GLOBOCAN 2020, we estimated the 2002–2019 cancer deaths by applying multiples of the age-specific mortality rates for 2020 to the corresponding age-specific annual female population sizes extracted from the United Nations World Population Prospects^37^. We considered four scenarios (S) for the multiples of the 2020 age-specific mortality rates as follows: (S1) Stable mortality rates (λ_y_ in year y) throughout 2002–2020 that is, λ_y_=λ_2020_; (S2) Mortality rates increased by 1% every year during 2002–2020, thus λ_y_= 0.99^(2020-y)^λ_2020_; (S3) Mortality rates decreased by 1% every year during 2002–2020, thus λ_y_=1.01^(2020-y)^λ_2020_; (S4) Mortality rate changes depended on region, with increasing mortality rates (scenario 2) for low and middle HDI countries and decreasing mortality rates (scenario 3) for high and very high HDI countries. Combined with the varying mortality rate ratios (MRR) due to being MC-orphans (MRR_MC_ above), the main prevalence estimates are provided for scenarios S1a and S2–4a/e as sensitivity analyses. (Extended Data Table 3).

All analyses were performed in R (version 4.1.2). All estimates were rounded to the nearest thousand when reported in the text.

This analysis was not submitted for institutional ethical approval as all data sources used are publicly available aggregate-level estimates.

### Reporting summary

Further information on research design is available in the Nature Portfolio Reporting Summary linked to this article.

## Online content

Any methods, additional references, Nature Portfolio reporting summaries, source data, extended data, supplementary information, acknowledgements, peer review information, details of author contributions and competing interests, and statements of data and code availability are available at 10.1038/s41591-022-02109-2.

### Supplementary information

Reporting Summary

Supplementary Table 1 Number of new and prevalent maternal orphans due to cancer, 2020, by country.

## Data Availability

The data sources are all publicly available datasets: The number of female cancer deaths in 2020 was sourced from IARC, GLOBOCAN (https://gco.iarc.fr/today/). They are available for 185 countries worldwide. Fertility and mortality rates were extracted from the 2019 revision of the United Nations World Population Prospect (UN-WPP; https://population.un.org/wpp/), which are available for 201 countries/territories with at least 90,000 inhabitants and included the 185 countries for which cancer deaths are available. The country-specific estimates of maternal orphans due to cancer are all provided in Supplementary Table 1.

## Extended data

**Extended Data Table 1:** New maternal orphans in 2020 (number, number per 100 cancer deaths in women, number per 100,000 children) for all cancers and for 14 major cancers by level of HDI

	Low HDI countries				Medium HDI countries				High HDI countries				Very High HDI countries			
	No.	%	No. per 100 cancer deaths in women	No. per 100,000 children	No.	%	No. per 100 cancer deaths in women	No. per 100,000 children	No.	%	No. per 100 cancer deaths in women	No. per 100,000 children	No.	%	No. per 100 cancer deaths in women	No. per 100,000 children
New maternal orphans due to all cancer deaths	280,310	27	118	55	319,241	30	48	37	308,072	29	16	36	129,026	12	8	35
New maternal orphans by type of cancer-related death -t																
Breast	79,919	28.5	147	15.7	73,593	23.1	53	8.6	65,847	21.4	26	7.8	34,866	27.0	15	9.6
Cervical	77,560	27.7	143	15.3	67,326	21.1	61	7.9	47,857	15.5	37	5.6	14,852	11.5	34	4.1
Upper Gastrointestinal	26,877	9.6	85	5.3	47,300	14.8	40	5.5	46,308	15.0	9	5.5	14,383	11.1	5	4.0
Hematological	18,114	6.5	106	3.6	27,116	8.5	57	3.2	27,307	8.9	24	3.2	8,627	6.7	7	2.4
Other female specific	12,924	4.6	84	2.5	18,872	5.9	31	2.2	21,180	6.9	18	2.5	9,442	7.3	7	2.6
Lower Gastrointestinal	11,632	4.1	94	2.3	11,150	3.5	43	1.3	18,490	6.0	10	2.2	11,246	8.7	6	3.1
Respiratory	3,397	1.2	63	0.7	10,687	3.3	32	1.3	23,671	7.7	8	2.8	12,364	9.6	5	3.4
Brain and nervous system	3,770	1.3	111	0.7	10,203	3.2	64	1.2	14,203	4.6	27	1.7	6,364	4.9	16	1.8
Head and Neck	5,596	2.0	94	1.1	18,695	5.9	42	2.2	7,782	2.5	21	0.9	2,381	1.8	10	0.7
Genitourinary	4,430	1.6	72	0.9	2,825	0.9	28	0.3	3,172	1.0	8	0.4	2,172	1.7	4	0.6
Skin cancer	2,352	0.8	62	0.5	2,025	0.6	37	0.2	2,334	0.8	14	0.3	2,546	2.0	10	0.7
Kaposi Sarcoma	5,863	2.1	204	1.2	2,547	0.8	182	0.3	531	0.2	85	0.1	22	0.0	10	0.0
Thyroid	1,378	0.5	66	0.3	1,335	0.4	31	0.2	1,850	0.6	14	0.2	503	0.4	6	0.1
Other	26,496	9.5	114	5.2	25,570	8.0	49	3.0	27,540	8.9	21	3.2	9,258	7.2	7	2.5

HDI: Human Development Index; No: Number of new maternal orphans due to cancer in 2020. 10 of 185 countries/territories are not included as they do not have a HDI value. They represent 10,000 new maternal orphans due to cancer in 2020.

**Extended Data Table 2:** Range of plausible estimates of global maternal orphan due to cancer point prevalence in mid-2020

	2002–2019 GLOBOCAN cancer mortality rates (MR) in women as a function of 2020 rates	Mortality Rate Ratios in children associated with being a maternal orphan				
		1.25	1.5	2	2.6	1
Scenario		a	b	c	d	e
1	Stable MR=MR_2020_	7,048,905*	7,030980	6,995,799	6,954,709	7,067,062
2	Increasing MR=MR_2020/_/ 1.01^(2020-Year)	6,701,135	6,684,748	6,652,573	6,614,974	6,717,728
3	Decreasing MR=MR_2020_ x 1.01^(2020-Year)	7,422,926	7,403,297	7,364,789	7,319,840	7,442,813
4**	Region-specific MR	6,917,011	6,900,619	6,868,421	6,830,770	6,933,603

*Estimates from Scenario 1a are our best-estimates and thus are the central estimates provided in the text; **For scenario 4, we applied scenario 2 cancer mortality rates for low and middle HDI countries/territories and scenario 3 rates to high and very high HDI countries.

MR: Mortality rates.

**Extended Data Table 3:** List of the 35 topographical cancer sites and their groupings

No.	Cancer groups	Cancer sites (ICD 10)
1	Brain	Brain, nervous system (C70–72)
2	Breast	Breast (C50)
3	Cervical	Cervix uteri (C53)
4	Genitourinary	Kidney and renal pelvis (C64–65)
5		Bladder (C67)
6	Hematological	Hodgkin lymphoma (C81)
7		Non-Hodgkin lymphoma (C82–86, C96)
8		Multiple myeloma and immunoproliferative diseases (C88+C90)
9		Leukemia (C91–95)
10	Head and Neck	Lip, oral cavity (C00–06)
11		Salivary glands (C07–08)
12		Oropharynx (C09–10)
13		Nasopharynx (C11)
14		Hypopharynx (C12–13)
15		Larynx (C32)
16	Kaposi sarcoma	Kaposi sarcoma (C46)
17	Lower Gastrointestinal	Colon (C18)
18		Rectum (C19–20)
19		Anus (C21)
20	Other	Other specified sites (C17, C24, C30–31, C37-38, C40-41,C47-49,C57-58,C63,C66,C69,C74-75)
21		Unspecified sites (C76-80+C97)
22	Other female specific	Vulva (C51)
23		Vagina (C52)
24		Corpus uteri (C54)
25		Ovary (C56)
26	Respiratory	Trachea, bronchus and lung (C33-34)
27		Mesothelioma (C45)
28	Skin cancer	Melanoma of skin (C43)
29		Non-melanoma skin cancer (C44)
30	Thyroid	Thyroid (C73)
31	Upper Gastrointestinal	Esophagus (C15)
32		Stomach (C16)
33		Liver and intrahepatic bile ducts (C22)
34		Gallbladder (C23)
35		Pancreas (C25)

ICD-10: International Classification of Disease 10th revision

## Code availability

All R codes used for data preparation and analysis are publicly available at https://code.iarc.fr/guidaf/maternalcancerorphans.
