# Supplementary material for: Global and regional estimates of orphans attributed to maternal cancer mortality in 2020
Source: Nat Med. 2022 Nov 20;28(12):2563–72. doi: 10.1038/s41591-022-02109-2 (PMC9676732; doi:10.1038/s41591-022-02109-2)
Supplement: Supplementary file 1 — Reporting Summary [file 41591_2022_2109_MOESM1_ESM.pdf]

nature portfolio

Corresponding author(s): Florence Guida and Valerie McCormack

Last updated by author(s): Oct 25, 2022

Reporting Summary

Nature Portfolio wishes to improve the reproducibility of the work that we publish. This form provides structure for consistency and transparency in reporting. For further information on Nature Portfolio policies, see our Editorial Policies and the Editorial Policy Checklist.

Statistics

For all statistical analyses, confirm that the following items are present in the figure legend, table legend, main text, or Methods section.

n/a	Confirmed	
☐	☒	The exact sample size (*n*) for each experimental group/condition, given as a discrete number and unit of measurement
☐	☒	A statement on whether measurements were taken from distinct samples or whether the same sample was measured repeatedly
☒	☐	The statistical test(s) used AND whether they are one- or two-sided<br>*Only common tests should be described solely by name; describe more complex techniques in the Methods section.*
☐	☒	A description of all covariates tested
☐	☒	A description of any assumptions or corrections, such as tests of normality and adjustment for multiple comparisons
☐	☒	A full description of the statistical parameters including central tendency (e.g. means) or other basic estimates (e.g. regression coefficient) AND variation (e.g. standard deviation) or associated estimates of uncertainty (e.g. confidence intervals)
☒	☐	For null hypothesis testing, the test statistic (e.g. *F*, *t*, *r*) with confidence intervals, effect sizes, degrees of freedom and *P* value noted<br>*Give P values as exact values whenever suitable.*
☒	☐	For Bayesian analysis, information on the choice of priors and Markov chain Monte Carlo settings
☒	☐	For hierarchical and complex designs, identification of the appropriate level for tests and full reporting of outcomes
☒	☐	Estimates of effect sizes (e.g. Cohen's *d*, Pearson's *r*), indicating how they were calculated

Our web collection on statistics for biologists contains articles on many of the points above.

Software and code

Policy information about availability of computer code

Data collection	No software was used for data collection.
Data analysis	All data were analysed using R version 4.1.2 (2021-11-01). All R codes used for data preparation and analysis are publicly available at https://code.iarc.fr/guidaf/maternalcancerorphans.

For manuscripts utilizing custom algorithms or software that are central to the research but not yet described in published literature, software must be made available to editors and reviewers. We strongly encourage code deposition in a community repository (e.g. GitHub). See the Nature Portfolio guidelines for submitting code & software for further information.

Data

Policy information about availability of data

All manuscripts must include a data availability statement. This statement should provide the following information, where applicable:

- Accession codes, unique identifiers, or web links for publicly available datasets
- A description of any restrictions on data availability
- For clinical datasets or third party data, please ensure that the statement adheres to our policy

The data sources are all publicly available datasets: The number of female cancer deaths in 2020 were sourced from IARC, GLOBOCAN (https://gco.iarc.fr/today/). They are available for 185 countries worldwide. Fertility and mortality rates were extracted from the 2019 revision of the United Nations World Population Prospect

(UN-WPP; https://population.un.org/wpp/) which are available for 201 countries/territories with at least 90,000 inhabitants and included the 185 countries for which cancer deaths are available. The country-specific estimates of maternal orphans due to cancer are all provided in the Supplementary Table 1.

Human research participants

Policy information about studies involving human research participants and Sex and Gender in Research.

Reporting on sex and gender	N/A
Population characteristics	N/A
Recruitment	N/A
Ethics oversight	N/A

Note that full information on the approval of the study protocol must also be provided in the manuscript.

Field-specific reporting

Please select the one below that is the best fit for your research. If you are not sure, read the appropriate sections before making your selection.

☐ Life sciences ☒ Behavioural & social sciences ☐ Ecological, evolutionary & environmental sciences

For a reference copy of the document with all sections, see nature.com/documents/nr-reporting-summary-flat.pdf

Behavioural & social sciences study design

All studies must disclose on these points even when the disclosure is negative.

Study description	Our study is a descriptive study based on publicly available data. Maternal orphans due to cancer were estimated by multiplying IARC Globocan estimates of age-country-specific cancer deaths in women in 2020 (absolute numbers; quantitative) with the UN-WPP average fertility of the corresponding female birth cohort since mid-2002 (quantitative), accounting for child mortality prior to maternal death (also from UN-WPP; quantitative).
Research sample	To estimate maternal orphan due to cancer, we needed country-level data on three items: (a) the absolute number of female cancer deaths in 2020, (b) fertility rates in women during 2002-2019, and (c) mortality rates of children during 2003-2020. We used all the publicly available data, i.e. number of cancer deaths in 2020 for the 185 countries available in Globocan. UN-WPP fertility estimates were extracted for women aged 15 to 49 years (outside of which we assumed that fertility is zero).
Sampling strategy	We included all countries/territories worldwide for which both fertility data and numbers of cancer deaths were available. The data sources are the most comprehensive UN databases for each type of data.
Data collection	The data sources are all publicly available. Estimates of the number of female cancer deaths in 2020 were sourced from IARC, GLOBOCAN. In brief, the GLOBOCAN estimates are assembled at the national level using the best available sources of cancer incidence and mortality data within a given country, with priority given to short-term mortality predictions (doi:10.1002/ijc.33588). Fertility and mortality rates were extracted from the 2019 revision of the United Nation World Population Prospect (UN-WPP; https://population.un.org/wpp/) which are available for 201 countries/territories with at least 90,000 inhabitants.
Timing	We used Globocan female cancer deaths recorded between 1 January 2020 and 31 December 2020 and UN-WPP fertility and mortality data recorded for each potential year of birth of the child i.e between 1 July 2002 and 30 June 2020.
Data exclusions	No data were excluded from the analyses.
Non-participation	Our study is a descriptive study based on publicly available data, there was no recruitment of single individuals.
Randomization	Our study is a descriptive study thus no randomization was performed.

Reporting for specific materials, systems and methods

We require information from authors about some types of materials, experimental systems and methods used in many studies. Here, indicate whether each material, system or method listed is relevant to your study. If you are not sure if a list item applies to your research, read the appropriate section before selecting a response.

Materials & experimental systems

n/a	Involved in the study
☒	☐ Antibodies
☒	☐ Eukaryotic cell lines
☒	☐ Palaeontology and archaeology
☒	☐ Animals and other organisms
☒	☐ Clinical data
☒	☐ Dual use research of concern

Methods

n/a	Involved in the study
☒	☐ ChIP-seq
☒	☐ Flow cytometry
☒	☐ MRI-based neuroimaging
